# Hematopoietic stem cell transplantation for DOCK8 deficiency: durable immune reconstitution and long-term survival in 47 patients

**DOI:** 10.3389/fimmu.2026.1930996

**Published:** 2026-09-10

**Authors:** Bashayr Alanazi, Reem Mohammed, Ali Al-Ahmari, Nora Alrumayan, Hawazen Alsaedi, Mouhab Ayas, Sultan Albuhairi, Sahar Elshorbagi, Rand Arnaout, Anas M. Alazami, Bandar AlSaud, Hamoud Al-Mousa

**Affiliations:** 1 Section of Pediatric Allergy and Immunology, Department of Pediatrics, King Faisal Specialist Hospital and Research Centre, Riyadh, Saudi Arabia; 2 Section of Stem Cell Transplantation, Department of Pediatric Hematology/Oncology, King Faisal Specialist Hospital and Research Centre, Riyadh, Saudi Arabia; 3 Department of Translational Genomics, Genomic Medicine Centre of Excellence, King Faisal Specialist Hospital and Research Center, Riyadh, Saudi Arabia

**Keywords:** combined immunodeficiency, dedicator of cytokinesis 8, DOCK8 deficiency, hematopoietic stem cell transplantation, inborn error of immunity

## Abstract

**Background:**

DOCK8 deficiency is a severe autosomal recessive combined immunodeficiency characterized by recurrent infections, severe atopy, immune dysregulation, and an increased risk of malignancy. Allogeneic hematopoietic stem cell transplantation (HSCT) remains the only curative treatment. We aimed to evaluate the long-term survival, immune reconstitution, and clinical outcomes of patients with DOCK8 deficiency following HSCT.

**Methods:**

We conducted a retrospective single-center study of 47 patients with genetically confirmed DOCK8 deficiency who underwent HSCT at King Faisal Specialist Hospital and Research Centre between 2012 and 2025. Clinical, immunologic, genetic, transplant-related, and survival outcomes were analyzed.

**Results:**

Eczema was present in all patients, while 91.5% had food allergies and 55% had recurrent sinopulmonary infections. Pre-transplant malignancy was identified in 17% of patients. Genetic analysis revealed predominantly homozygous loss-of-function variants. HSCT was performed at a median age of 6 years, using matched related donors in 37/47 patients (79%), matched unrelated donors in 7/47 (15%), haploidentical donors in 2/47 (4%), and an umbilical cord blood graft in 1/47 (2%). After a median follow-up of 7 years, overall survival and event-free survival were both 91.5%. Survival was 94.6% following matched related donor transplantation and 100% following matched unrelated donor transplantation. Mortality was limited to the early post-transplant period and was primarily associated with pre-existing malignancy or irreversible organ damage. All survivors demonstrated complete resolution of eczema and recurrent infections and robust immune reconstitution.

**Conclusions:**

HSCT provides excellent long-term survival and durable immune correction in patients with DOCK8 deficiency. Early diagnosis and transplantation before the development of irreversible organ damage are critical determinants of favorable outcomes.

## Introduction

Dedicator of Cytokinesis 8 (DOCK8) deficiency is a severe combined immunodeficiency caused by biallelic pathogenic variants in the *DOCK8* gene. First described in 2009, DOCK8 deficiency was initially classified as autosomal recessive hyper-IgE syndrome, but it is currently recognized as a distinct entity separate from the autosomal dominant form caused by STAT3 defects based on its unique clinical and immunological features (1, 2). The disorder is characterized by recurrent and often severe bacterial, viral, and fungal infections, with a particular predisposition to cutaneous viral infections such as herpes simplex virus (HSV), varicella-zoster virus, human papillomavirus (HPV), and molluscum contagiosum, alongside prominent atopic manifestations including eczema, food allergies, asthma, and allergic rhinitis, typically accompanied by markedly elevated serum IgE levels and eosinophilia. In addition to infectious susceptibility, patients exhibit significant immune dysregulation, manifesting as autoimmunity, chronic inflammation, and impaired vaccine responses. The disease follows a progressive course, with cumulative organ damage caused by recurrent infections and persistent viral disease often leading to complications, such as bronchiectasis and chronic lung disease, and it is associated with a markedly increased risk of malignancy, particularly virus-driven cancers including squamous cell carcinoma and lymphomas (3–5). Patient prognosis is poor without curative treatment, with mortality approaching 50% by the second decade of life (3).

The cellular and immunologic bases of DOCK8 deficiency are rooted in the critical role of the DOCK8 protein in regulating actin cytoskeletal dynamics and lymphocyte migration. DOCK8 is essential for preserving lymphocyte structural integrity during migration through dense tissue environments, and its absence leads to defective trafficking and impaired survival of T cells, B cells, and natural killer (NK) cells as they traverse anatomical barriers. In addition, DOCK8 deficiency results in profound abnormalities in lymphocyte differentiation and function, characterized by CD4^+^ and CD8^+^ T-cell lymphopenia, reduced invariant natural killer T (iNKT) and mucosal-associated invariant T (MAIT) cell populations, skewing toward Th2 polarization, and impaired regulatory T-cell function. Collectively, these immunologic defects compromise antiviral immunity and immune regulation, predisposing affected individuals to persistent and widespread viral infections and markedly increasing the lifetime risk of cutaneous and hematologic malignancies (6–8).

Allogeneic hematopoietic stem cell transplantation (HSCT) is the only curative therapy for DOCK8 deficiency. Replacement of defective hematopoietic stem cells with donor-derived cells restores immune competence and corrects the underlying immunologic defect. Accumulating evidence from large international cohorts indicates that HSCT provides substantial clinical benefits, including resolution of recurrent infections and significant improvement or complete remission of eczema, although certain atopic manifestations might persist in some patients (9, 10). These findings are supported by multicenter collaborative studies, including an international cohort of 81 patients that reported an overall survival (OS) rate of 84% after median follow-up of 26 months, with improved outcomes observed in patients receiving reduced-toxicity conditioning regimens (11). Similarly, in a prospective study of 36 patients with DOCK8 deficiency undergoing HSCT reported high efficacy with successful engraftment, sustained full donor chimerism, and an OS rate of 80.6% after median follow-up of 7.4 years (12). Beyond clinical outcomes, mechanistic studies have confirmed that HSCT restores lymphocyte development and function, including normalization of T- and B-cell compartments and improved immune synapse formation, thereby directly correcting the cellular defects underlying DOCK8 deficiency (13). Collectively, these data support early referral for HSCT as the standard of care to prevent irreversible organ damage, reduce malignancy risk, and improve long-term survival.

In this study, we report the largest single-center experience to date of hematopoietic stem cell transplantation (HSCT) in patients with genetically confirmed DOCK8 deficiency. Over a 13-year period, 47 patients underwent HSCT and were followed for a median of 7 years, demonstrating excellent long-term survival, durable immune reconstitution, and sustained resolution of disease manifestations. Our findings also provide important insights into donor selection, conditioning strategies, transplant-related complications, and long-term clinical outcomes.

## Methods

### Patients

This retrospective cohort study included patients with genetically confirmed DOCK8 deficiency managed at the Immunodeficiency Clinics of King Faisal Specialist Hospital and Research Centre (KFSHRC; Riyadh, Saudi Arabia) between August 2012 and April 2025. Data extracted from institutional medical records and transplant databases included demographics, clinical manifestations, immunologic and genetic findings, details of HSCT procedures, post-transplant complications, immune reconstitution, and clinical outcomes at the latest follow-up.

The study protocol was approved by the Institutional Review Board of KFSHRC (RAC: 2251867; approved December 30, 2025). In accordance with institutional policies and international ethical standards, written informed consent for the use and publication of potentially identifiable clinical data and images was obtained from all patients or their legal guardians.

### Genetic testing

Genomic DNA was extracted from peripheral blood leukocytes using standard methods. Genetic analysis was performed using next-generation sequencing (NGS) approaches, including targeted primary immunodeficiency gene panels or whole-exome sequencing, depending on availability and time of diagnosis. Libraries were prepared using standard Illumina (San Diego, CA, USA) protocols and sequenced on the NovaSeq platform (Illumina). Sequence reads were aligned to the human reference genome, followed by variant calling and annotation as previously described (14, 15).

DOCK8 variants were prioritized according to their consistency with the clinical phenotype and autosomal recessive mode of inheritance. To detect copy number variations (CNVs), particularly large deletions involving *DOCK8*, chromosomal microarray analysis on a high-resolution single-nucleotide polymorphism-based platform was performed in selected patients. Regions of homozygosity were also assessed, especially in the context of consanguinity. CNVs were interpreted according to size, gene content, and overlap with known genomic variants using publicly available databases, including DGV, ClinVar, and DECIPHER. The pathogenicity of identified variants was evaluated according to the American College of Medical Genetics and Genomics guidelines, incorporating *in silico* prediction tools, conservation analysis, and previously reported data. All clinically significant variants were confirmed by Sanger sequencing, and segregation analysis was performed in available family members when feasible.

### Preparative regimen, transplantation, and supportive care

The enrolled patients underwent HSCT at a median age of 6 years (interquartile range [IQR], 3–10). High-resolution molecular human leukocyte antigen (HLA) typing was performed for class I (HLA-A, HLA-B, HLA-C) and class II (HLA-DRB1, HLA-DQB1) loci. Matched sibling donors (MSDs; n = 29) were prioritized whenever available, followed by matched family donors (MFDs; n = 8), matched unrelated donors (MUDs; n = 7), haploidentical parental donors (n = 2), and an unrelated umbilical cord blood (UCB) (n = 1). Stem cell sources were unmanipulated bone marrow (n = 39), peripheral blood stem cells (n = 7), and UCB (n = 1).

Conditioning regimens were individualized according to donor type, transplant era, and the patient’s clinical condition. Busulfan/cyclophosphamide (Bu/Cy) was used predominantly before 2015 and was subsequently largely replaced by busulfan/fludarabine (Bu/Flu). Treosulfan/fludarabine (Treo/Flu) was used in selected patients in whom a reduced-toxicity conditioning approach was considered preferable based on pretransplant clinical status and the anticipated risk of regimen-related toxicity, with the aim of achieving adequate engraftment while minimizing treatment-related toxicity. Among recipients of matched related donor (MRD) grafts, 9 patients received Bu/Cy, 24 received Bu/Flu, and 2 received Treo/Flu. Two additional patients received BEAM conditioning (carmustine, etoposide, cytarabine, and melphalan) because of residual lymphoma detected on pretransplant positron emission tomography (PET). Rabbit antithymocyte globulin (ATG) was administered to 16 patients. Intravenous busulfan was administered every 6 hours for 4 consecutive days (16 doses), on days −10 to −7 in the Bu/Cy regimen and days −6 to −3 in the Bu/Flu regimen. Weight-based dosing was used: 1.0 mg/kg/dose for patients weighing <9 kg, 1.2 mg/kg/dose for 9–<16 kg, 1.1 mg/kg/dose for 16–23 kg, 0.95 mg/kg/dose for >23–34 kg, and 0.8 mg/kg/dose for >34 kg. Pharmacokinetic monitoring and AUC-guided busulfan dose adjustment were not routinely performed during the study period. Cyclophosphamide was administered at 50 mg/kg/day on days −5 to −2, fludarabine at 30 mg/m²/day on days −6 to −2, and treosulfan at 10–14 g/m²/day on days −5 to −3, with treosulfan dosing determined according to body surface area (BSA). ATG was administered at 2 mg/kg/day on days −5 to −2. All matched unrelated donor (MUD) recipients received Bu/Flu/ATG conditioning. Of the two haploidentical recipients, one received Bu/Flu/thiotepa/ATG, with thiotepa administered at 10 mg/kg on day −5, whereas the other received Treo/Flu/ATG. The umbilical cord blood (UCB) recipient received Bu/Cy/ATG conditioning.

The median infused CD34^+^ cell dose for MRD, MUD, and haploidentical transplants was 6.78 × 10^6^ cells/kg (IQR, 4.35–9.00 × 10^6^ cells/kg). For the UCB transplant, the infused CD34^+^ cell dose was 0.09 × 10^6^ cells/kg, with a total nucleated cell (TNC) dose of 1.25 × 10^8^ cells/kg. Graft-versus-host disease (GVHD) prophylaxis consisted primarily of cyclosporine A (CSA), initiated on day −3 at 3 mg/kg/day and subsequently dose-adjusted to maintain a target trough concentration of 150–250 ng/mL. For matched related donor and matched unrelated donor (MUD) transplants, CSA was combined with short-course methotrexate (MTX) at 10 mg/m² on days +1, +3, and +6. In matched related donor recipients, CSA was gradually tapered after day +100, with the timing of discontinuation individualized according to GVHD status, donor chimerism, and overall clinical status. In MUD recipients, CSA was tapered after day +100 with the aim of discontinuation by approximately day +180 in the absence of GVHD. The two recipients of maternal matched family donor grafts who received treosulfan/fludarabine (Treo/Flu) conditioning additionally received abatacept at 10 mg/kg on days −1, +5, +14, and +28 as intensified GVHD prophylaxis to reduce donor T-cell activation and the risk of acute GVHD. Haploidentical transplant recipients received post-transplant cyclophosphamide (PTCy) on days +3 and +4, followed by CSA and mycophenolate mofetil (MMF) from day +5. MMF was discontinued on day +35 in the absence of GVHD, while CSA was continued through day +180 and subsequently tapered in the absence of GVHD. The umbilical cord blood (UCB) recipient received CSA and corticosteroids for GVHD prophylaxis.

All patients were managed in positive-pressure isolation rooms throughout the transplantation period. During the aplastic phase, antimicrobial prophylaxis included pentamidine for Pneumocystis jirovecii pneumonia (PJP) prophylaxis, with subsequent transition to trimethoprim-sulfamethoxazole (TMP-SMX) following hematologic recovery; intravenous acyclovir (1,500 mg/m²/day) for herpes simplex virus (HSV) and varicella-zoster virus (VZV) prophylaxis, with subsequent transition to oral acyclovir following hematologic recovery and when oral administration was feasible; and voriconazole for antifungal prophylaxis. Febrile neutropenia was managed according to the institutional protocol with intravenous piperacillin-tazobactam and gentamicin as initial empirical antibacterial therapy, with subsequent modification according to clinical status, microbiological findings, and antimicrobial susceptibility results.

Viral surveillance for cytomegalovirus (CMV), Epstein–Barr virus (EBV), and adenovirus was performed weekly by quantitative polymerase chain reaction (PCR) through day +100 after HSCT. Monitoring was continued beyond day +100 in patients with ongoing risk factors, including active GVHD, continued immunosuppressive therapy, delayed immune reconstitution, or prior viral reactivation. According to the institutional protocol, pre-emptive CMV therapy with ganciclovir or foscarnet was generally initiated at a CMV viral load ≥1,000 copies/mL, although treatment could be initiated at lower levels in high-risk patients or in the setting of rapidly increasing DNAemia. For EBV, initiation of pre-emptive therapy was guided by the magnitude and kinetics of EBV DNAemia together with individual risk factors for post-transplant lymphoproliferative disease (PTLD), rather than by a single predefined viral-load threshold. When indicated, pre-emptive therapy consisted primarily of rituximab, together with reduction of immunosuppression when clinically feasible. Supportive care included transfusion of leukoreduced and irradiated blood products, with CMV-seronegative components used when clinically indicated according to institutional transfusion policy. As part of the institutional supportive-care protocol, granulocyte colony-stimulating factor (G-CSF) was administered at 5 μg/kg/day from day +7 until the absolute neutrophil count (ANC) exceeded 0.5 × 10^9^/L for 3 consecutive days.

Intravenous immunoglobulin (IVIG) replacement therapy was administered every 3–4 weeks, with the dose adjusted according to clinical status and to maintain an IgG trough level ≥8 g/L. Discontinuation of IVIG was individualized based on evidence of humoral immune reconstitution, including B-cell recovery, endogenous immunoglobulin production, and the absence of recurrent or severe infections. Post-HSCT revaccination was initiated following discontinuation of IVIG and evidence of adequate immune reconstitution, starting with inactivated vaccines. Functional humoral recovery was assessed by measuring vaccine-specific antibody responses to pneumococcal and tetanus vaccines approximately 8 weeks after immunization. Live attenuated vaccines were considered from 24 months post-HSCT in patients who demonstrated satisfactory immune reconstitution and were free of active GVHD and systemic immunosuppressive therapy.

### Assessment of engraftment, GVHD and immune reconstitution

Neutrophil engraftment was defined as the first of 3 consecutive days with an absolute neutrophil count (ANC) ≥0.5 × 10^9^/L. Platelet engraftment was defined as the first day on which the platelet count reached ≥20 × 10^9^/L and remained above this threshold without platelet transfusion for at least 7 consecutive days. Donor chimerism was assessed by short tandem repeat (STR)-based analysis at approximately days +30, +60, and +100, at 6 months and 1 year, and annually thereafter. Complete donor chimerism was defined as ≥95% donor-derived cells and mixed chimerism as 5% to <95% donor-derived cells. Lineage-specific donor chimerism was assessed separately in the lymphoid and myeloid lineages. Acute GVHD was graded according to the modified Glucksberg criteria and chronic GVHD according to the National Institutes of Health (NIH) consensus criteria, with histopathologic confirmation obtained when clinically indicated. First-line treatment of clinically significant GVHD consisted of systemic corticosteroids, with additional immunosuppressive or immunomodulatory therapies introduced according to disease severity and response to initial treatment (16, 17).

Immune reconstitution was evaluated longitudinally by lymphocyte subset enumeration, serum immunoglobulin levels, and T-cell proliferative responses to phytohemagglutinin (PHA). PHA responses were expressed as a percentage of the concurrent healthy control response and categorized according to the performing laboratory’s reference criteria as severely depressed (<30%), markedly depressed (30–49%), moderately depressed (50–64%), mildly depressed (65–75%), or normal (>75%).

### Outcome definitions

OS was defined as the time from HSCT to death from any cause, with surviving patients censored at the time of last follow-up. Event-free survival (EFS) was defined as the time from HSCT to the occurrence of the first event, including graft failure, need for a second transplantation, or death from any cause. Patients without events were censored at the last follow-up.

### Statistical analysis

Continuous variables were summarized as medians and IQRs, and categorical variables were summarized as counts and percentages. OS and EFS were estimated using the Kaplan–Meier method. Meanwhile, 95% confidence intervals (CIs) were calculated using Greenwood’s formula with log-log transformation. Comparisons between strata were performed using the two-sided log-rank test, with p < 0.05 considered statistically significant.

## Results

### Patient characteristics and clinical presentation

In total, 47 patients from 32 unrelated families with genetically confirmed DOCK8 deficiency underwent allogeneic HSCT, including two patients previously reported in a multicenter cohort study (3). Parental consanguinity was documented in all families. The cohort included 24 girls (51%) and 23 boys (49%). Thirteen patients were diagnosed through targeted screening based on a known familial history. The median age at symptom onset was 12 months (IQR 5–36); the median age at diagnosis was 36 months (IQR 16.5–63), corresponding to a median diagnostic delay of 12 months (IQR 4–28). All patients presented with eczema (100%). Food allergy was documented in 43 patients (91.5%), of whom 13 (30.2%) had experienced anaphylaxis. Other atopic manifestations included asthma in 24 patients (51%) and allergic rhinitis in 4 (8.5%). Recurrent sinopulmonary infections were observed in 55% of patients, while skin abscesses (42.5%) and otitis media (36%) were also common. Bacterial infections were frequent, with methicillin-resistant *Staphylococcus aureus* (MRSA) documented in 47% of patients, in addition to infections caused by Gram-negative organisms. Fungal infections included Candida species (30%) and Pneumocystis jirovecii (12%). Viral manifestations included herpes simplex virus type 1 (HSV-1) infection in 11 patients (23.4%), molluscum contagiosum in 8 (17%), and human papillomavirus (HPV)-associated warts in 5 (10.6%). Cytomegalovirus (CMV) and Epstein–Barr virus (EBV) DNAemia were documented in 24 (54%) and 16 (37.5%) of patients, respectively.

Eight patients (17%) had a history of malignancy prior to transplantation. These included EBV-associated adrenal smooth muscle tumors (n = 2), EBV-driven lymphomas with central nervous system involvement (n = 2), endobronchial leiomyoma (n = 1), Burkitt lymphoma (n = 1), perianal squamous cell carcinoma (n = 1), and chronic eosinophilic leukemia (n = 1). Each affected patient had a single malignancy, with no patient developing more than one malignant condition. The frequencies of the major clinical manifestations are summarized in **Table 1**.

**Table 1:** The frequencies of major clinical manifestations among the 47 patients with DOCK8 deficiency.

Clinical feature	Number (%)
Eczema	47 (100%)
Food allergy	43 (91.5%)
Anaphylaxis	13 (30.2%)
Asthma	24 (51%)
Allergic rhinitis	4 (8.5%)
Sinopulmonary infections	26 (55%)
Skin abscesses	20 (42.5%)
Otitis media	17 (36%)
HSV-1	11 (23.4%)
Molluscum contagiosum	8 (17%)
HPV-warts	5 (10.6%)
Malignancy	8 (17%)
Bronchiectasis	7 (15%)
Chronic diarrhea	7 (15%)
Liver disease	6 (12.7%)
Eye disease (keratitis/conjunctivitis)	6 (12.7%)
Autoimmune hemolytic anemia.	2 (4%)

### Laboratory and immunologic characteristics

Pretransplant evaluation revealed peripheral eosinophilia in all patients, with a median eosinophil count of 8 x 10^9^/L, (range, 2.6–29.8 x 10^9^/L). Age-adjusted immunophenotyping demonstrated CD4^+^ T-cell lymphopenia in 31 patients (IQR 367-799) and CD8^+^ T-cell reduction in 25 patients (IQR 224-679). NK cell lymphopenia was observed in 14 patients (IQR 57-421), whereas B-cell lymphopenia was less frequent, affecting eight patients (IQR 521-1722) (18). T-cell function was assessed in 23 patients using lymphocyte proliferation assays in response to phytohemagglutinin (PHA), demonstrating severe to marked impaired responses ranging from 5% to 42% of those observed in healthy controls.

Serum immunoglobulin analysis revealed hypogammaglobinemia, including reduced IgG levels in 20 patients (42%), and reduced IgM levels in 42 patients (89%) while serum IgA was low in 2 patients (4.5%). Marked IgE elevation was a consistent feature, with a median level of 11,890 kU/L (IQR, 2,596–34,260).

### Genetic defects

**Figure 1:**
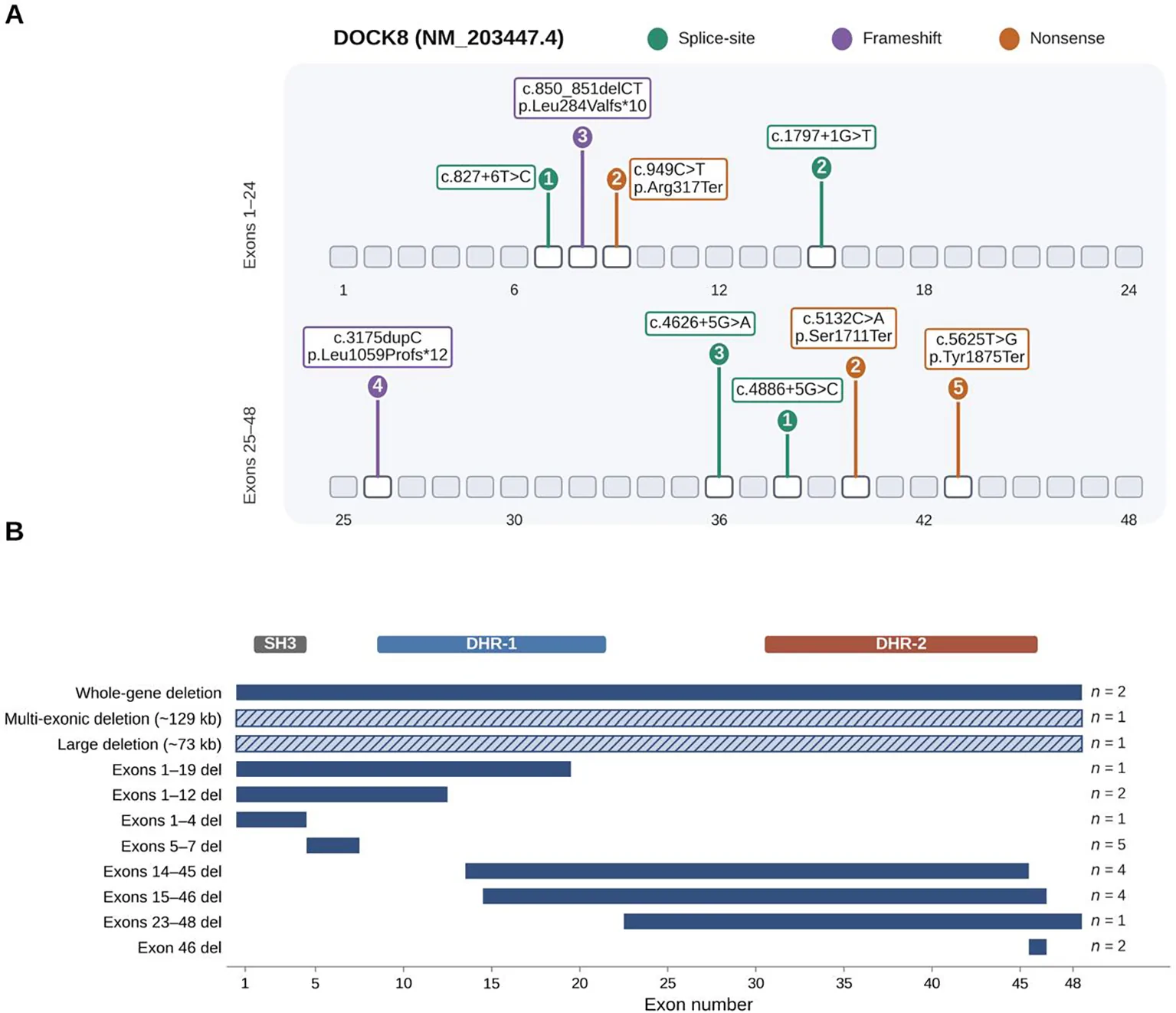
Spectrum of *DOCK8* variants identified in the study cohort. **(A)** Distribution of pathogenic DOCK8 variants across the 48 exons of transcript NM_203447.4. Circular lollipops indicate variant locations, and numbers denote the number of patients harboring each variant. Colors represent mutation class (splice-site, frameshift, and nonsense). Exons are not drawn to genomic scale. **(B)** Distribution of large DOCK8 deletions relative to the SH3, DHR-1, and DHR-2 protein domains. Bars represent the extent of each deletion; hatched bars indicate deletions with incompletely defined breakpoints. Numbers on the right indicate the number of patients carrying each deletion.

Molecular analysis identified homozygous pathogenic *DOCK8* variants (NM_203447.4) in all 47 patients, consistent with the autosomal recessive inheritance pattern. The mutational spectrum was dominated by loss-of-function variants, including large multi-exonic or whole-gene deletions (51%), nonsense variants (19%), frameshift variants (15%), and splice-site variants (15%) All identified variants are summarized in **Figure 1** and **Table 2**. **Figure 1A** demonstrates that pathogenic small variants are distributed across the DOCK8 gene without a clear mutational hotspot, whereas **Figure 1B** illustrates the marked heterogeneity of structural variants, ranging from focal exon deletions to large multi-exonic and whole-gene deletions, including deletions involving regions encoding the DHR-1 and DHR-2 functional domains.

**Table 2:** DOCK8 pathogenic variants identified among the 47 patients.

Variant	Zygosity	Nucleotide change	Protein change	Number	Reported
Deletion	Homozygous	Exons 1–4 del	–	1	Yes
	Homozygous	Exons 1–12 del	–	2	Yes
	Homozygous	Exons 1–19 del	–	1	Yes
	Homozygous	Exons 5–7 del	–	5	Yes
	Homozygous	Exons 14–45 del	–	4	Yes
	Homozygous	Exons 15–46 del	–	4	Yes
	Homozygous	Exons 23–48 del	–	1	Yes
	Homozygous	Exon 46 del	–	2	Yes
	Homozygous	Multi-exonic deletion	–	2	Yes
	Homozygous	Whole gene deletion	–	2	Yes
Nonsense	Homozygous	c.5132C>A	p.Ser1711Ter	2	Yes
	Homozygous	c.949C>T	p.Arg317Ter	2	Yes
	Homozygous	c.5625T>G	p.Tyr1875Ter	5	Yes
Frameshift	Homozygous	c.850_851delCT	p.Leu284Valfs*10	3	Yes
	Homozygous	c.3175dupC	p.Leu1059Profs*12	4	Yes
Splice-site	Homozygous	c.4626 + 5G>A	–	3	Yes
	Homozygous	c.4886 + 5G>C	–	1	No
	Homozygous	c.1797 + 1G>T	–	2	Yes
	Homozygous	c.827 + 6T>C	–	1	Yes

Most variants identified in our cohort have been previously reported and they are known to result in loss of DOCK8 protein expression. In addition, we identified a novel homozygous splice-site variant, c.4886 + 5G>C, in one patient. This variant affects the canonical donor splice site of intron 38, and RT-PCR analysis of patient-derived mRNA confirmed exon 38 skipping. The aberrant transcript is predicted to generate a premature termination codon, leading to nonsense-mediated mRNA decay and the consequent absence of functional DOCK8 protein. Importantly, this truncation would result in loss of the downstream DHR2 (DOCKER) domain, which is critical for CDC42 binding and guanine nucleotide exchange factor activity, thereby supporting a loss-of-function mechanism.

### HSCT engraftment, survival, and toxicity

**Figure 2:**
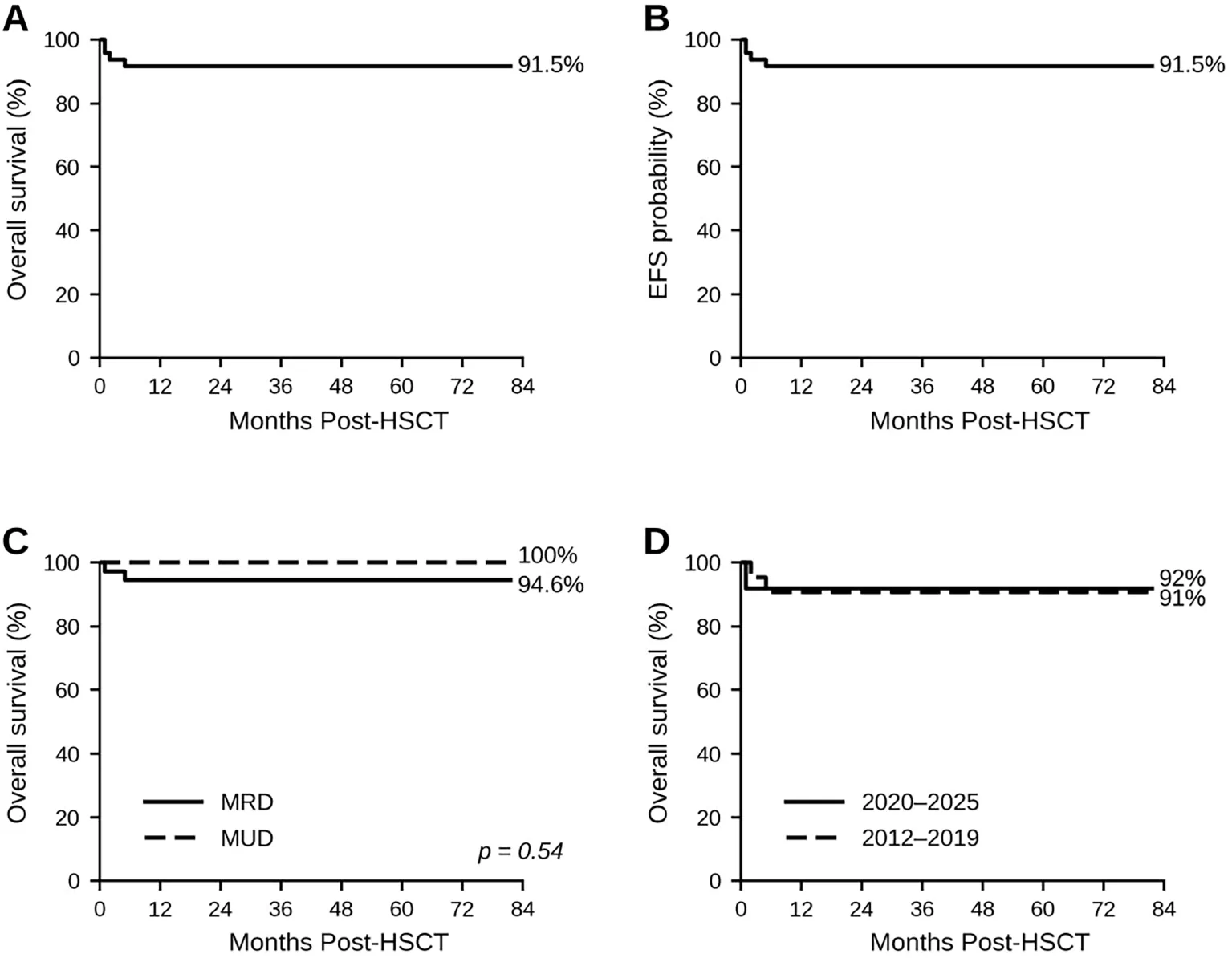
Kaplan–Meier survival outcomes following HSCT for DOCK8 deficiency. **(A)** Overall survival (OS) of the entire cohort. **(B)** Event-free survival (EFS) of the entire cohort. **(C)** OS according to donor type, comparing matched related donor (MRD) and matched unrelated donor (MUD) transplantation. **(D)** OS according to transplant era (2012–2019 vs 2020–2025).

After median follow-up of 7 years (IQR 3.3–10.2), the OS and EFS rates were both 91.5% (95% CI, 78.9%–96.7%; **Figures 2A, B**). **Figure 2A** demonstrates an estimated OS of 91.5% for the entire cohort. All deaths occurred within the first 5 months after HSCT, after which the survival curve remained stable throughout long-term follow-up. **Figure 2B** demonstrates an EFS of 91.5%, identical to OS, reflecting the absence of additional qualifying events such as graft failure or second transplantation among surviving patients. **Figure 2C** showed that survival did not differ significantly between donor types (p = 0.54), OS rates of 94.6% (95% CI, 80.1%–98.6%) and 100% for MRDs and MUDs, respectively. Finally, **Figure 2D** demonstrates similarly favorable survival in the 2012–2019 and 2020–2025 transplant eras (approximately 92% and 91%, respectively), indicating consistently favorable outcomes across the study period. Survival outcomes by donor category are presented in in **Table 3**. Four patients died, including three patients with pre-existing malignancy, and two patients with significant irreversible end-organ damage, including pneumatocele-associated bronchiectasis and chronic liver disease prior to transplantation. The causes of death were primarily related to sepsis, acute respiratory distress syndrome, and multiorgan failure. CMV reactivation occurred in six patients during the early post-transplant period, and two of the deaths were associated with viral disease. Notably, no deaths occurred beyond 5 months after transplantation (**Table 4**).

**Table 3:** Patients and transplant characteristics among the 47 patients.

Characteristic	n	Survival n (%)
Sex		
Female	24	22 (91.6%)
Male	23	21 (91.3%)
Age (years) at HSCT, median (range)	6 (1–17)	
Donor type		
Matched sibling donor	29	27 (93%)
Matched family donor	8	8 (100%)
Matched unrelated donor	7	7 (100%)
Haploidentical	2	1 (50%)
Umbilical cord blood	1	0
Stem cell source		
Bone marrow	39	36 (92.3%)
Peripheral blood stem cells	7	7 (100%)
Cord blood	1	0
Conditioning		
Busulfan-based	42	40 (95.2%)
Busulfan/cyclophosphamide	10	8 (80%)
Busulfan/fludarabine	32	32 (100%)
Treosulfan/fludarabine	3	2 (66.7%)
Other (BEAM)	2	1 (50%)
Antithymocyte globulin serotherapy	26	24 (92.3%)

HSCT, hematopoietic stem cell transplantation; BEAM, carmustine, etoposide, cytarabine, and melphalan conditioning.

**Table 4:** Clinical and transplant characteristics of deceased patients.

Patient	Sex	Age at HSCT	Donor	Conditioning	Pre-transplant status	Chimerism at last follow up	Day of death	Cause of death (clinical course)
D1	F	14 y	MSD	Bu/Cy	Perianal squamous cell carcinoma	L 100% M 100%	Day +150	CMV colitis, EBV reactivation,fungal pneumonia and septic shock
D2	F	7 y	Cord blood (UCB)	Bu/Cy/ATG	Recurrent infections, pneumatocele	Not done	Day +17	Graft failure, CMV viremia, polymicrobial pneumonia and refractory septic shock
D3	M	10 y	Haploidentical	Treo/Flu/ATG	Chronic eosinophilic leukemia; cystic bronchiectasis; chronic liver disease	Not done	Day +35	ARDS / pulmonary hemorrhage, septic shock and hepatic failure
D4	M	4 y	MSD	BEAM	EBV-driven CNS high-grade B-cell lymphoma (stage IV)	Not done	Day +12	Hemorrhagic shock, encephalopathy and multiorgan failure

Neutrophil engraftment was achieved in 46 patients, with a median time to neutrophil recovery of 18 days (IQR, 16–20). The median time to platelet engraftment was 19 days (IQR, 18–25). Only one case of primary graft failure occurred, involving the UCB recipient, while no secondary graft failure was observed in the cohort. GVHD occurred in 12 patients (25.5%). Acute GVHD was observed in 7 patients; all cases were grade I–II, with skin and/or gastrointestinal involvement. Two patients had isolated gastrointestinal involvement, 4 had isolated skin involvement, and 1 had combined skin and gastrointestinal involvement. No grade III–IV acute GVHD occurred. Chronic GVHD developed in 5 patients, including mild skin-limited disease in 3 patients and ocular involvement in 2. No severe chronic GVHD was observed. All patients with GVHD responded to standard therapy. Veno-occlusive disease (VOD) occurred in one patient who received busulfan/fludarabine conditioning and was successfully treated with defibrotide. Despite the relatively high prevalence of pretransplant EBV DNAemia (34%), biopsy-confirmed post-transplant lymphoproliferative disease (PTLD) developed in only two patients. Both achieved complete remission following rituximab therapy and reduction of immunosuppression. Complete donor chimerism in both myeloid and lymphoid lineages was achieved in 42/43 surviving patients. One patient exhibited stable mixed chimerism but demonstrated excellent clinical recovery with sustained normalization of immune function. Outcomes across the different HSCT donor types are summarized in **Table 5**.

**Table 5:** Overall survival, engraftment and GVHD incidence among various HSCT donor types.

Donor type		MRD	MUD	Haploidentical	UCB
Patients (n)		37	7	2	1
Neutrophils engraftment, median (range)		18 (8–37)	18 (17-28)	22	–
Platelet engraftment, median (range)		19 (15-45)	18 (17-21)	22	–
Primary graft failure		–	–	–	1
Donor chimerism at day +30					
Lymphoid, (range) % Complete lymphoid chimerism, n Mixed lymphoid chimerism, n Myeloid, (range) Complete myeloid chimerism, n Mixed myeloid chimerism, n		66-100 21 15 95-100 34 2	74-100 4 3 95-100 6 1	74 - 1 100 1 -	–
Donor chimerism at the last follow-up					
Lymphoid, (range) % Complete lymphoid chimerism, n/N Mixed lymphoid chimerism, n Myeloid, (range) % Complete myeloid chimerism, n/N Mixed myeloid chimerism, n		97-100 35 0 94-100 34 1	100 7 - 100 7 -	100 1 - 100 1 -	–
GVHD prophylaxis					
	CSA/MTX	37	7	–	–
	CSA/MMF	–	–	2	–
	CSA/Steroid	-	–	–	1
	Abatacept	2	–	–	–
	PT/CY		–	2	
Acute GVHD		7	–	–	–
Chronic GVHD		5	–	–	–
Transplant-related mortality		2	–	1	1
Surviving patients, n (%)		35 (94.6%)	7 (100%)	1 (50%)	0

MRD, matched related donor; MUD, matched unrelated donor; UCB, umbilical cord blood; GVHD, graft-versus-host disease; CSA, cyclosporine; MTX, methotrexate; MMF, mycophenolate mofetil; PT/CY, post-transplant cyclophosphamide

### Immune reconstitution

Following HSCT, all the 43 surviving patients exhibited marked clinical and immunologic improvement. After median follow-up of 7 years, the median lymphocyte subset counts were as follows: CD3^+^ T cells, 1851 cells/mm³ (IQR 1568-2479); CD4^+^ T cells, 1037 cells/mm³ (IQR 732-1316); CD8^+^ T cells, 788 cells/mm³ (IQR 609-1048); CD19^+^ B cells, 689 cells/mm³ (IQR 522-757); and CD56^+^/CD16^+^ NK cells, 226 cells/mm³ (IQR 127-286). In patients who underwent functional assessment, lymphocyte proliferative responses to PHA were substantially improved, ranging from 64% to 191% of healthy control values. IVIG replacement therapy was successfully discontinued in all 43 survivors at a median of 8 months after HSCT. All patients subsequently developed protective antibody responses following routine immunization. A significant reduction in serum IgE levels (median 188 kU/L) and normalization of IgG, IgM and IgA levels were also observed.

### Clinical outcome after HSCT

All surviving patients exhibited marked clinical improvement following HSCT. Eczema resolved completely in all 43 survivors. Among the 39 evaluable patients with pre-transplant food allergy, 20 patients (51.3%) achieved complete resolution of food allergy, while 19 patients (48.7%) continued to have persistent food allergy. Recurrent bacterial and viral skin infections resolved within 3–6 months after HSCT. No secondary malignancies were observed during the follow-up period among survivors. Post-transplant autoimmunity developed in four patients. One patient had suspected systemic lupus erythematosus, which was well controlled with hydroxychloroquine. Alopecia areata occurred in one patient and remained stable with topical therapy. Thyroid autoimmunity consistent with Graves’ disease was identified in two patients, both of whom achieved stable disease control with antithyroid therapy (methimazole).

## Discussion

The clinical phenotype of our cohort was highly consistent with the well-established spectrum of DOCK8 deficiency. All patients presented with eczema, and the majority had food allergies, reflecting the prominent Th2-skewed immune response that defines this disorder. The frequency of atopic manifestations in our cohort was comparable to that reported in large international series, in which eczema and allergic disease were observed in approximately 80–90% of patients (3–5, 9). Infectious complications were also a major component of the disease burden. Recurrent sinopulmonary infections, skin abscesses, and viral infections were frequently observed, consistent with previous reports (3–5). The rate of viral skin disease in our cohort was lower than that described in earlier studies, which might reflect earlier diagnosis and improved supportive care, potentially limiting progression to extensive viral involvement. Pre-transplant systemic viral reactivation, including CMV and EBV, was common, underscoring the profound impairment in antiviral immunity associated with DOCK8 deficiency (6–8). The observed rate of pretransplant malignancy was in line with previously reported frequencies of 10–20% (3, 4). The predominance of virus-associated cancers, particularly EBV-driven lymphoproliferative disease and HPV-related tumors, underscores the pivotal role of impaired immune surveillance in the pathogenesis of DOCK8 deficiency. In addition, the presence of complications such as bronchiectasis, chronic liver disease, and gastrointestinal involvement reflects the cumulative burden of persistent infection and inflammation over time. Collectively, these findings highlight the progressive nature of the disease and reinforce the importance of early diagnosis and timely intervention before the onset of irreversible organ damage.

The genetic architecture of DOCK8 deficiency in our cohort was predominated by homozygous loss-of-function variants, with large multi-exonic deletions representing the most frequent genetic defects. This observation is consistent with prior findings, and it reflects both the genomic instability of the *DOCK8* locus and the high prevalence of consanguinity in our population (1, 3). *DOCK8* spans a large genomic region on chromosome 9p24.3, predisposing it to structural rearrangements, including deletions that might not be reliably detected by standard sequencing approaches. Consequently, our findings underscore the critical importance of incorporating CNV analysis, such as chromosomal microarray or depth-of-coverage algorithms in NGS pipelines, into the routine diagnostic evaluation of suspected cases (15). In addition to large deletions, a spectrum of truncating variants, including nonsense, frameshift, and splice-site mutations, was identified, with all variants predicted to result in complete loss of protein function. This is consistent with the established disease mechanism, whereby DOCK8 deficiency arises from absent or severely reduced protein expression. Although genotype–phenotype correlations in DOCK8 deficiency remain incompletely defined, the predominance of null variants across different cohorts suggests that most patients share a broadly similar and severe clinical phenotype.

HSCT produced excellent long-term outcomes concerning both OS and EFS in our cohort. The recorded outcomes compare favorably with those in previously published studies, which reported survival rates of approximately 80–84% with shorter follow-up durations (11, 12). These favorable outcomes should, however, be interpreted in the context of the predominance of matched related donor transplantation and the relatively young median age at HSCT (6 years) of our cohort. The high proportion of matched related donors may have contributed to the favorable survival and transplant-related outcomes and may limit the generalizability of our findings to settings where alternative donors are more frequently required. Similarly, transplantation at a younger age may have allowed HSCT to be performed before the development of advanced infectious, malignant, or irreversible organ complications associated with DOCK8 deficiency. As a key observation of our study, mortality was largely confined to the early post-transplant period, occurring predominantly in patients with advanced pre-existing disease, including malignancy and irreversible organ damage. No late mortality was observed, underscoring the durability of transplant success once early complications are overcome. These findings strongly support early referral for HSCT, ideally before the development of significant comorbidities.

Notably, outcomes were particularly favorable among patients receiving matched donor transplants, in line with prior reports (11, 12). Although outcomes with alternative donors, such as haploidentical and cord blood transplantation, were less favorable, the small sample size limits definitive conclusions. The conditioning approach evolved over the study period, with Bu/Cy used predominantly in the earlier era and subsequently replaced largely by Bu/Flu, while Treo/Flu was reserved for selected patients in whom a reduced-toxicity approach was considered preferable. Although survival and engraftment outcomes were favorable across conditioning approaches, the small and markedly unbalanced subgroups, particularly the limited number of Treo/Flu recipients, precluded meaningful comparison of conditioning-related outcomes. The clinical impact of HSCT was substantial, as all survivors experienced complete resolution of eczema and cessation of recurrent infections within months of transplantation. Immune reconstitution was robust, with normalization of lymphocyte subsets, improved functional responses, and successful discontinuation of IVIG replacement in most patients. These findings confirm that HSCT corrects the underlying immunologic defect in DOCK8 deficiency. Transplant-related toxicity was acceptable, with low rates of severe complications. Acute GVHD was limited to mild events, and severe acute or extensive chronic GVHD was not observed. The low incidence of PTLD, despite high pre-transplant EBV exposure, reflects effective monitoring and early intervention strategies. However, autoimmune manifestations persisted or developed in a small subset of patients, suggesting that immune dysregulation might not be fully reversed in all patients.

A notable observation in our cohort was the differential response of atopic manifestations following HSCT. While eczema resolved completely in all surviving patients, food allergy persisted in nearly half of evaluable patients (48.7%) despite successful donor engraftment and immune reconstitution. This finding is consistent with previous reports demonstrating that food allergy may persist after HSCT in patients with DOCK8 deficiency, even following myeloablative conditioning and full donor chimerism (19). The mechanisms underlying this phenomenon remain incompletely understood but may involve the survival of long-lived recipient-derived allergen-specific IgE-producing plasma cells within nonhematopoietic niches, persistence of tissue-resident memory responses, or delayed re-establishment of immune tolerance to dietary antigens. In contrast, eczema appears to be more directly dependent on the underlying immune dysregulation characteristic of DOCK8 deficiency and therefore responds more readily to correction of the hematopoietic compartment. These findings have important clinical implications, indicating that although HSCT effectively cures the infectious complications and eczematous disease associated with DOCK8 deficiency, food allergy may persist long term. Accordingly, continued allergy follow-up, periodic reassessment of sensitization status, and cautious, supervised dietary reintroduction remain essential components of post-transplant care.

Although HSCT is the current standard curative therapy for DOCK8 deficiency, gene therapy represents a promising future alternative, particularly for patients lacking suitable donors or those at high risk of transplant-related complications. As a monogenic disorder, DOCK8 deficiency is theoretically well suited for gene correction strategies using autologous hematopoietic stem cells. Advances in gene therapy have achieved success in other inborn errors of immunity, including adenosine deaminase deficiency and Wiskott–Aldrich syndrome, providing a strong proof of concept for similar approaches in DOCK8 deficiency (20, 21). However, the large size of the DOCK8 gene presents technical challenges for conventional viral vector-based approaches. Emerging gene-editing technologies, such as CRISPR/Cas9, offer a potential solution by enabling the precise correction of pathogenic variants at the endogenous locus. Preclinical studies suggest that restoration of DOCK8 expression can rescue key cellular defects, including impaired lymphocyte survival and migration (22). Despite this promise, significant challenges remain, including optimizing delivery systems, ensuring long-term safety, and achieving durable multilineage engraftment. Although gene therapy is not yet clinically available for DOCK8 deficiency, ongoing advances in the field suggest that it could become a viable therapeutic option in the future.

This study had several limitations. Its retrospective, single-center design introduced the potential for selection bias and incomplete data capture, and the predominantly consanguineous population might limit generalizability. Although this was among the largest single-center cohorts of DOCK8 deficiency, subgroup analyses, particularly by donor type and conditioning regimen were limited by relatively small sample sizes. Additionally, the absence of a control group limited direct comparison with non-transplanted patients. The study strengths included its large cohort size, uniform management within a single specialized center, and extended long-term follow-up, permitting comprehensive assessments of clinical and immunologic outcomes.

Future research should aim to refine both diagnostic and therapeutic approaches for DOCK8 deficiency. Earlier detection through family-based screening might enable timely intervention and improve clinical outcomes. Well-designed prospective multicenter studies are needed to better define the optimal timing of HSCT and to evaluate outcomes across different stages of disease. In addition, efforts should focus on optimizing conditioning regimens, particularly in younger patients, to reduce toxicity while maintaining reliable and durable engraftment. Finally, long-term follow-up is critical to evaluate the sustainability of immune reconstitution and to monitor late complications such as autoimmunity and secondary malignancies.

## Conclusion

This study represents the largest single-center experience of HSCT for DOCK8 deficiency, with one of the longest reported follow-up periods. In this predominantly MRD-transplanted cohort, HSCT performed at a relatively young age was associated with excellent long-term survival, durable donor engraftment, sustained immune reconstitution, and complete resolution of recurrent infections and eczema. Favorable outcomes were also observed among MUD recipients, although the small number of patients in this subgroup limits definitive conclusions. Mortality was confined to the early post-transplant period and occurred predominantly in patients with advanced disease, supporting transplantation before the development of irreversible organ damage or malignancy. Although food allergy persisted in a subset of patients despite successful transplantation, overall clinical and immunological outcomes improved substantially. These findings reinforce HSCT as the established curative therapy for DOCK8 deficiency and underscore the importance of early diagnosis, family screening, and timely referral for transplantation. Further optimization of transplant strategies and emerging gene-based therapies may provide additional opportunities to improve outcomes in this severe inborn error of immunity.

## Data Availability

Data supporting the findings of this study are available from the corresponding author upon reasonable request, subject to institutional and ethical restrictions.
